# Alcohol’s Effects on Male Reproduction

**Published:** 1998

**Authors:** Mary Ann Emanuele, Nicholas V. Emanuele

**Affiliations:** Mary Ann Emanuele, M.D., is a professor in the Department of Medicine, the Department of Molecular and Cellular Biochemistry, and the Division of Research on Drugs of Abuse, Loyola University Stritch School of Medicine, Maywood, Illinois. Nicholas V. Emanuele, M.D., is a professor in the Department of Medicine, the Division of Research on Drugs of Abuse, and the Molecular Biology Program and director of the Division of Endocrinology and Metabolism at Loyola University Stritch School of Medicine, Maywood, Illinois, and a staff physician at the Veterans Affairs Hospital, Hines, Illinois

**Keywords:** AODE (alcohol and other drug effects), hypothalamus, pituitary gland, male genitals, reproductive function, testosterone, hormone metabolism, heavy AOD use, cell type, luteinizing hormone, follicle stimulating hormone, gonadotropin RH, secretion, animal model, male, literature review

## Abstract

The male reproductive system consists of the hypothalamus, the anterior pituitary gland, and the testes. Alcohol can interfere with the function of each of these components, thereby causing impotence, infertility, and reduced male secondary sexual characteristics. In the testes, alcohol can adversely affect the Leydig cells, which produce and secrete the hormone testosterone. Studies found that heavy alcohol consumption results in reduced testosterone levels in the blood. Alcohol also impairs the function of the testicular Sertoli cells that play an important role in sperm maturation. In the pituitary gland, alcohol can decrease the production, release, and/or activity of two hormones with critical reproductive functions, luteinizing hormone and follicle-stimulating hormone. Finally, alcohol can interfere with hormone production in the hypothalamus.

In both men and women, the hormones regulating reproduction form a complex and finely tuned system that affects virtually every cell system in the body. The male reproductive system consists of three parts: a brain region called the hypothalamus, the anterior pituitary (a gland that is located at the base of the brain but is not considered a brain region), and the testes. This article briefly reviews how those three organs and the hormones they produce cooperate to ensure and regulate male reproductive functioning. The article then describes alcohol’s effects on each of the three organs, drawing on studies in both humans and animals. The discussion also points out potential therapies for preventing or reversing alcohol’s deleterious effects.

## Overview of the Male Reproductive System

Of the three components of the male reproductive system, the hypothalamus and the anterior pituitary gland have solely regulatory functions, which are mediated by the hormones secreted from these two organs. The third component, the testes, also produces key hormones controlling male sexual characteristics and behaviors, the most important of which is testosterone. In addition, the testes are responsible for sperm production.

The hypothalamus, which is located at the base of the brain, is often called the master control unit of the reproductive systems of both men and women. (For more information on the hypothalamus and the hormones it produces, see the article by Hiller-Sturmhöfel and Bartke, pp. 153–164.) Among other hormones, the hypothalamus produces gonadotropin-releasing hormone (GnRH). GnRH is secreted in pulses into a system of blood vessels that connect the hypothalamus to the anterior pituitary gland, which is located just beneath the hypothalamus.

In response to the GnRH stimulus, the anterior pituitary gland produces two hormones that control reproductive functions—luteinizing hormone (LH) and follicle-stimulating hormone (FSH) —and releases them into the general circulation. Those two hormones have different functions in men and women. In men, LH stimulates the testes to produce the hormone testosterone, whereas FSH plays an important role in sperm maturation. (For information on the roles of LH and FSH in women, see the article by Hiller-Sturmhöfel and Bartke, pp.153–164.) In addition, the anterior pituitary gland produces the hormone prolactin. In men, elevated prolactin levels play a role in reproduction by indirectly suppressing testosterone levels in the body.^1^

**Figure:**
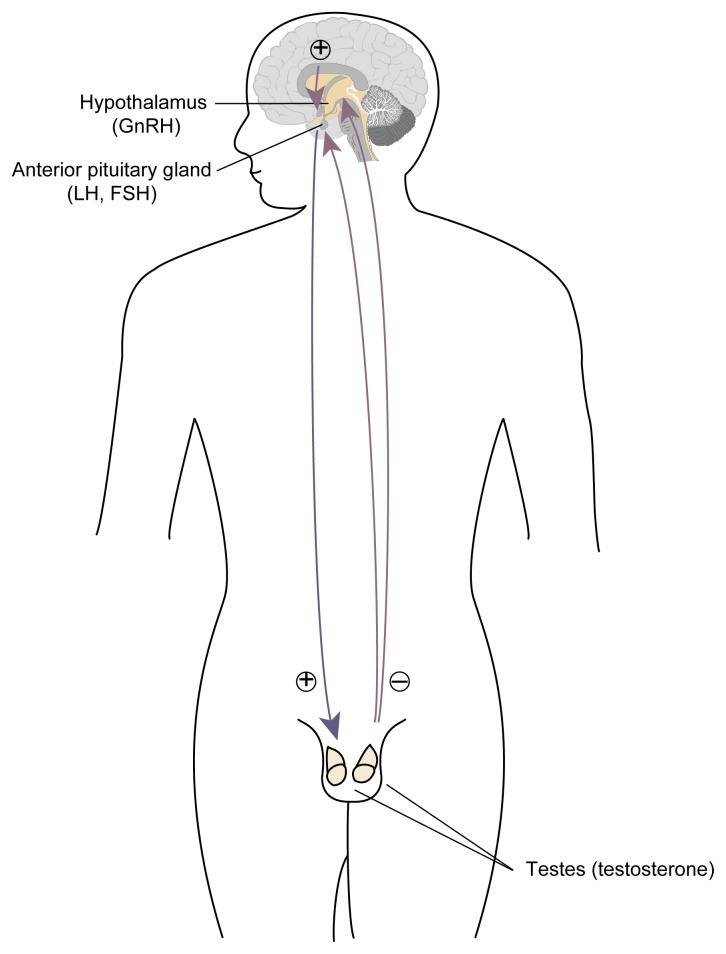
Components of the hypothalamic-pituitary-gonadal axis. The hypothalamus releases gonadotropin-releasing hormone (GnRH) to the anterior pituitary gland, which in turn releases luteinizing hormone (LH) and follicle-stimulating hormone (FSH) into the bloodstream. LH and FSH circulate to the testes, which release testosterone in response. When testosterone levels in the blood rise, the anterior pituitary becomes less responsive to GnRH stimulation; as a result, both LH and FSH secretion diminish and testosterone levels fall. This process is called a negative feedback mechanism. Likewise, when testosterone production declines, the anterior pituitary becomes more responsive to GnRH and increases its release of LH and FSH, thereby stimulating testosterone production. NOTE: ⊕ excites; ⊝ inhibits. Testosterone may also have a negative feedback effect on GnRH.

The testes consist primarily of the seminiferous tubules, the site of sperm cell formation and maturation. Interspersed among those tubules are so-called interstitial cells, including a cell type called Leydig cells, which produce the “male” sex hormone testosterone.^2^ In addition to its function in reproduction, testosterone helps regulate many diverse body functions, including bone and muscle development; red blood cell turnover; and development and maintenance of male sexual characteristics, such as sexual drive (i.e., libido), growth of facial and body hair, and deepening of the voice during puberty. Consequently, an insult to the hormonal system controlling testosterone production can result not only in infertility but also in other deleterious consequences, such as accelerated bone loss (i.e., osteoporosis), decreased muscle function, and lower than normal numbers of red blood cells (i.e., anemia).

A second important testicular cell type is the Sertoli cell. These cells, which are embedded in the inner walls of the seminiferous tubules, play a critical role in sperm development by supporting and nourishing the sperm cells during their maturation.

The three components of the male reproductive system—the hypothalamus, anterior pituitary, and testes—form a finely tuned system called the hypothalamic-pituitary-gonadal (HPG) axis, which is controlled through a classic negative feedback mechanism (see figure). As testosterone levels in the blood rise, the anterior pituitary becomes less responsive to stimulation by GnRH, resulting in reduced LH and FSH secretion. Because LH induces testosterone production, reduced LH secretion results in lowered testosterone levels. Conversely, if testosterone levels in the blood decline (e.g., because of an injury to the testes), the anterior pituitary’s responsiveness to GnRH increases and more LH and FSH are secreted, thereby promoting testosterone production by the Leydig cells. Some research findings suggest that testosterone also has a negative feedback function on GnRH.

In addition to this feedback loop, several hormones produced within and outside the testes regulate testosterone production. One hormone that is produced within the testes (as well as in other body regions, including the hypothalamus) is β-endorphin, a molecule similar to morphine (i.e., an endogenous opioid^3^). β-Endorphin produced within the testes suppresses testicular testosterone production and/or release. Furthermore, β-endorphin released in the hypothalamus results in decreased GnRH levels. Lower GnRH levels, in turn, lead to reduced LH and FSH secretion from the anterior pituitary and reduced testosterone production by the Leydig cells.

## Alcohol’s Effects on the Testes

Numerous studies have indicated that alcohol abuse in men can cause impaired testosterone production and shrinkage of the testes (i.e., testicular atrophy) (Adler 1992). Those changes can result in impotence, infertility, and reduced male secondary sexual characteristics (e.g., reduced facial and chest hair, breast enlargement, and a shift in fat deposition from the abdomen to the hip area). For example, in a classic study by Lloyd and Williams (1948), 72 percent of men with advanced alcoholic cirrhosis exhibited decreased libido and sexual potency. Similarly, Van Thiel and colleagues (1974) noted that impotence was “prominent in the majority of subjects, but occurred more frequently among patients with greater liver damage” (p. 1190). As a semiquantitative measure of the prevalence of impotence, those authors reported that of 22 men with cirrhosis, 18 men were unable to ejaculate for a semen analysis (i.e., were impotent), 2 men produced no sperm at all, 1 man had reduced sperm counts, and only 1 man had a normal sperm count.

Testicular atrophy also appears to be common among alcoholics, occurring in up to 75 percent of men with advanced alcoholic cirrhosis (Lloyd and Williams 1948). This atrophy likely is caused by several factors, including (1) alcohol’s damaging effects on the testes; (2) alcohol’s effects on LH and FSH (see the section “Alcohol’s Effects on the Anterior Pituitary Gland,” pp. 199–200), which, among other factors, stimulate testicular growth; and (3) various confounding factors, such as malnutrition, concomitant treatment with various medications, and abuse of drugs other than alcohol by the subjects. Testicular atrophy results primarily from the loss of sperm cells and decreased diameter of the seminiferous tubules (Van Thiel et al. 1974).

Finally, Lloyd and Williams (1948) reported that 87 percent of men with advanced alcoholic cirrhosis exhibited decreased amounts of axillary hair, whose development depends on testosterone and related hormones.

Subsequent studies have confirmed alcohol’s deleterious effects on the testosterone-producing Leydig cells, the Sertoli cells, and even on the offspring of alcohol-ingesting males (see textbox), independent of co-occurring liver disease or malnutrition.

**Paternal Alcohol Use and Fetal Development**

Paternal alcohol use may affect not only a man’s fertility but also the development of his offspring. Alcohol’s impact on fetal outcome is difficult to assess in humans, however, because confounding problems (e.g., other drug abuse or smoking) often are involved. Moreover, in many couples, not only the man but also his female partner abuses alcohol or other drugs, further complicating the analysis of the effects of paternal alcohol consumption on fetal development. As a result, the consequences of paternal alcohol ingestion on fertility and subsequent fetal abnormalities have been studied in rats. In those studies, male rats that had not been previously exposed to alcohol received a single moderate or high alcohol dose 24 hours before mating. Although alcohol administration did not affect mating behavior, it did result in reduced birth weights in the pups and reduced litter sizes (Cicero et al. 1994). Those findings suggest that acute paternal alcohol ingestion could adversely affect the outcome of the progeny. Clearly, this issue should be investigated further.

### Alcohol’s Effects on Leydig Cells and Testosterone Metabolism

Alcohol’s adverse effects on Leydig cell function and testosterone production were demonstrated in a study of young, healthy male volunteers with normal liver function who received alcohol over a 4-week period (Gordon et al. 1976). In that study, a 15-percent alcohol solution was administered every 3 hours, around the clock, together with a diet replete with protein, vitamins, folic acid, and minerals. The total daily alcohol dose was 220 grams, or approximately 3 grams per kilogram body weight. With this level of alcohol consumption, testosterone levels in the men’s blood declined as early as 5 days into the study and continued to fall over the entire study period. The investigators attributed the decline in testosterone to a decrease in the production rate and an increase in the breakdown and removal of testosterone from the blood (i.e., an increased metabolic clearance rate). Since those initial studies were performed, numerous studies in humans and laboratory animals have confirmed the reduction in testosterone levels after both one-time (i.e., acute) and long-term (i.e., chronic) alcohol exposure. For example, in healthy male rats a single alcohol dose resulted in a profound reduction in testosterone levels that lasted for up to 96 hours (Steiner et al. 1996).

Alcohol’s effects on testosterone metabolism are somewhat different, however, in men with alcoholic liver disease compared with men without alcoholic liver disease. Thus, although the production rates and blood levels of testosterone are reduced in both groups of men, the metabolic clearance of testosterone increases only in men without alcoholic liver disease. In men with alcoholic liver disease, in contrast, the metabolic clearance is decreased (Southren et al. 1973).

Another mechanism through which alcohol may lower testosterone levels is the conversion of testosterone or one of its precursors into estrogens through a process called aromatization. For example, testosterone can be metabolized to an estrogen called estradiol. Similarly, the immediate precursor of testosterone—androstenedione—can be converted into a less potent estrogen called estrone. This conversion process may be enhanced in men who regularly consume alcohol. Several studies found that some people with alcoholic liver disease have increased levels of estrogens in the blood (Van Thiel et al. 1974, 1978; Gordon et al. 1978). This increase does not appear to be caused by decreased estrogen breakdown and therefore must result from increased estrogen production (Gordon et al. 1978).

Animal studies have indicated that alcohol does not directly enhance estrogen production in the testes (which produce both testosterone and estrogen). Instead, increased aromatization of testosterone and androstenedione to estrogens occurs in other tissues, such as the liver and fat tissue (Gordon et al. 1979). In those tissues, alcohol stimulates an enzyme called aromatase, which mediates the aromatization reaction (Gordon et al. 1979). Consequently, in addition to decreased testosterone production and metabolism, higher-than-normal percentages of testosterone and androstenedione are converted into estradiol and estrone, respectively, in heavy drinkers. This increased conversion may account for the elevated estrogen levels and abnormal breast enlargement observed in some heavy drinkers. For example, in the study by Lloyd and Williams (1948), 42 percent of males with alcoholic cirrhosis exhibited enlarged breasts.

In addition to causing breast enlargement, estrogens appear to exert a negative feedback effect on LH and FSH production and may thereby contribute to alcohol’s suppression of those key reproductive hormones (see the section “Alcohol’s Effects on the Anterior Pituitary Gland”).

Clinical studies have demonstrated that alcohol not only alters testosterone metabolism but also diminishes testosterone production (e.g., Southren et al. 1973). To elucidate the mechanisms underlying the alcohol-induced reduction in testosterone secretion, researchers have investigated alcohol’s effects on testes studied outside the body (i.e., in vitro) or analyzed the testes independent of the rest of the body. In those experiments, testosterone production in the isolated testes decreased, as it had in studies in intact animals (e.g., Badr et al. 1977; Cobb et al. 1978) These findings indicate that alcohol exerts its effect, at least in part, by acting directly on the testes (although alcohol also affects hormone production in the hypothalamus and anterior pituitary, as described in the following sections).

Researchers have proposed several mechanisms that may contribute to the alcohol-induced testosterone suppression (Anderson et al. 1983). For example, investigators have suggested that alcohol’s breakdown product, acetaldehyde, may be a contributing factor, because in some studies acetaldehyde was more potent than alcohol in suppressing testosterone release (e.g., Badr et al. 1977; Cobb et al. 1978). Possibly, however, acetaldehyde does not itself suppress testosterone production. Instead, the enzyme that mediates the breakdown of alcohol to acetaldehyde uses certain molecules (i.e., cofactors) that are also required by enzymes involved in testosterone production, thereby preventing testosterone generation (Ellingboe and Varanelli 1979; Gordon et al. 1980).

Other studies have noted an increase in β-endorphin levels in the testicular fluid after acute alcohol exposure (Adams and Cicero 1991). As described previously, testicular β-endorphin inhibits testosterone production and/or release. Researchers recently confirmed the role of β-endorphin through a study in which rats were treated with a substance that inhibits β-endorphin activity (i.e., naltrexone) (Emanuele et al. 1998). In that study, naltrexone prevented the fall in testosterone after both acute and chronic (i.e., for 14 days) alcohol ingestion. Naltrexone, which is currently used in alcoholism treatment to decrease alcohol craving, therefore may potentially be used to prevent reductions in testosterone levels and the associated adverse consequences in alcoholics who are unable to discontinue drinking on their own.

Disturbances in other hormonal systems also may contribute to the alcohol-induced suppression of testosterone levels. For example, the adrenal hormones cortisol (in humans) and corticosterone (in rats) can suppress the reproductive system by inhibiting the ability of the Leydig cells to produce and release testosterone. Studies in humans and animals found that alcohol exposure increases adrenal hormone levels, thereby interfering with reproductive functions (Rivier and Vale 1988).

Finally, nitric oxide (NO), a gas found in all tissues, may contribute to alcohol’s toxic effects. NO affects numerous biological processes, including widening of the blood vessels (i.e., vasodilation), the immune response, communication between cells of the nervous system, and hormone secretion. For example, NO has been shown to decrease testosterone secretion (McCann and Rettori 1996). In the testes (as well as in many other tissues), the gas is generated by an enzyme called nitric oxide synthase (NOS). Research has indicated that inhibition of the enzyme NOS by various substances can prevent the alcohol-induced decline in testosterone levels (Adams et al. 1993; Shi et al. 1998). This observation suggests that alcohol and NO may enhance each other’s ability to decrease testosterone production. Accordingly, future strategies aimed at preventing or reversing alcohol-induced suppression of testicular function may include NOS inhibition.

### Alcohol’s Effects on Sertoli Cells

Sertoli cells also may be an important target for alcohol’s actions on the reproductive system. Researchers have observed sperm abnormalities in men with histories of moderate or heavy alcohol consumption. For example, in an autopsy study (Pajarinen et al. 1996), men with a history of low alcohol consumption (i.e., 10 to 40 grams, or approximately 1 to 3.5 standard drinks,^4^ per day) generally showed no abnormal sperm forms. Moderate alcohol consumption (i.e., 40 to 80 grams, or approximately 3.5 to 7 standard drinks, per day) was associated with a slight alteration in sperm maturation. Finally, a history of heavy alcohol consumption (more than 80 grams, or more than 7 drinks, per day) led to arrested sperm development in 20 percent of the cases. Studies in alcoholics who had not yet developed severe liver damage (i.e., in whom liver damage itself had not affected testicular function) found that 40 percent of the men studied had reduced sperm counts, 45 percent showed abnormal sperm shapes, and 50 percent exhibited altered sperm motility (Villalta et al. 1997).

The mechanisms underlying alcohol’s effects on the Sertoli cells have not yet been fully elucidated. It appears, however, that alcohol may damage some of the proteins required for sperm cell production that the Sertoli cells provide (Zhu et al. 1997).

## Alcohol’s Effects on the Anterior Pituitary Gland

### Effects on LH Production, Secretion, and Activity

With the development of sophisticated techniques that allow measuring even low hormone levels in the blood (i.e., radioimmunoassays), researchers now are able to assess changes in the levels of the pituitary hormones LH and FSH after alcohol exposure. Those studies confirmed an alcohol-related fall in testosterone levels. Surprisingly, however, the levels of LH in the blood often did not increase and, in fact, declined in some studies (van Thiel et al. 1974; Gordon et al. 1976, 1978; Ching et al. 1988; Adams and Cicero 1991; Emanuele et al. 1991, 1992). Those results were unexpected, because if alcohol exclusively affected the testes, the reduced testosterone levels should have evoked a rise in LH levels as a result of the feedback mechanism regulating the HPG axis. Accordingly, the lack of an increase in LH levels in many cases implied that alcohol acted not only on the testes but also on the hypothalamus and/or the pituitary gland. Subsequently, studies in alcohol-fed rats established that the decrease in LH blood levels resulted from impairments in both LH production and LH secretion (Emanuele et al. 1991).

A related concern is whether alcohol affects LH release at the level of the pituitary and/or the hypothalamus. To address this issue, researchers have removed anterior pituitary glands from animals and grown them in vitro in the presence or absence of alcohol (Pohl et al. 1987; Emanuele et al. 1989). The results of those experiments suggest that alcohol can decrease LH secretion even from isolated pituitary glands, implying that alcohol lowers LH levels at least in part by acting directly on the pituitary.

Scientists have attempted to identify the specific step(s) in LH production and secretion that are impaired by alcohol. LH production is initiated by the interaction of GnRH released from the hypothalamus with specific docking molecules (i.e., receptors) on the surface of those pituitary cells that are involved in LH generation and secretion. That interaction activates a cascade of enzymes in those cells. Conceivably, alcohol could disrupt the functioning of the GnRH receptor or its interaction with GnRH, thereby leading to diminished LH release. To date, however, scientists have found no evidence indicating that the interaction of GnRH with its receptor is impaired.

Accordingly, alcohol probably interferes with one or more events that happen within the cell after GnRH has attached to its receptor. Researchers have identified one such reaction. For GnRH to stimulate the production and release of LH effectively, an enzyme called protein kinase C must move from within the LH-producing cell to the cell’s surface. Alcohol has been shown to prevent this movement of protein kinase C (Steiner et al. 1997). The chain of events from the binding of GnRH to the pituitary cell to the release of LH, however, is highly complex. Consequently, alcohol probably also interferes with other steps in this process. The identification of those steps will lead to a more complete understanding of how alcohol disrupts pituitary function.

In addition to reducing LH levels in the blood, alcohol also may affect LH activity. Like many other hormones, LH is not a simple protein but a glycoprotein—that is, a protein to which various sugar molecules are attached. The number and types of sugar molecules attached to the protein portion determine the hormone’s biological potency (i.e., its ability to stimulate testosterone production). Numerous LH variants differing in their attached sugar molecules exist that differ substantially in their potencies. Alcohol has been shown to result in the production of less potent LH variants (Emanuele et al. 1986). Thus, alcohol’s deleterious effects on LH function are qualitative as well as quantitative.

### Effects on FSH Production and Activity

Research also has indicated that alcohol reduces FSH levels in the blood, although this effect is not as consistent as its effect on LH levels (van Thiel et al. 1974, 1978; Gordon et al. 1976, 1978; Salonen and Huhtaniemi 1990; Emanuele et al. 1992). As mentioned earlier in this article, FSH influences the activity of the testicular Sertoli cells, which support sperm cell development and maturation. Both reduced numbers of sperm and abnormal sperm forms have been found in men with histories of heavy drinking. Those changes in sperm formation may be associated with the decreased fertility reported in alcohol-abusing men. Furthermore, in light of the alcohol-induced disruption of Sertoli cell function and reduction in testosterone levels, one would expect FSH levels to be elevated, because FSH is part of the same negative feedback mechanism as LH. Consequently, “normal” FSH levels actually should be considered too low for the accompanying testosterone levels.

## Alcohol’s Effects on the Hypothalamus

Alcohol may affect LH levels by acting not only directly on the pituitary gland but also on the hypothalamus. For example, one study in male rats found that alcohol administration significantly lowered GnRH levels in the blood vessels connecting the hypothalamus to the pituitary gland (Ching et al. 1988). Subsequent investigations using isolated hypothalami from male rats or GnRH-producing cells obtained from genetically engineered mice, however, failed to demonstrate any reduction in GnRH secretion in response to alcohol treatment (Emanuele et al. 1990; Uddin et al. 1996). Moreover, researchers detected no alcohol-induced reduction in the activity (i.e., expression) of the gene that is responsible for generating GnRH, suggesting that alcohol probably does not affect GnRH production (Uddin et al. 1996). Together, the available evidence suggests that alcohol decreases GnRH secretion by acting at a site outside the hypothalamus and/or that alcohol’s breakdown products (e.g., acetaldehyde), rather than alcohol itself, reduces GnRH secretion. The former option appears highly likely, because GnRH secretion is controlled by a complex mechanism involving various nerve impulses generated outside the hypothalamus. Alcohol might act on any of those impulses.

In addition to interfering with GnRH secretion, alcohol also appears to affect the production of active GnRH molecules. Like many protein hormones, GnRH first is produced as a large, inactive precursor molecule called pre-pro-GnRH, which consists of 92 building blocks (i.e., amino acids). To generate the functional GnRH molecule, 82 of those amino acids are removed from the inactive precursor molecule. This processing reaction from an inactive precursor to an active molecule appears to be diminished after alcohol exposure (Uddin et al. 1996).

## Conclusions

Alcohol can have profound deleterious effects at all levels of the male reproductive system. Although researchers have learned much about those effects in the past two decades, many questions remain unanswered. For example, future research must focus on the effects of alcohol ingestion earlier in life (e.g., during the prepubertal and pubertal years) when reproductive organs, particularly the testes, are maturing and may be more susceptible to alcohol-induced damage. Such investigations are particularly relevant in light of the fact that many teenagers consume alcohol, although it is illegal for them to do so. In addition, researchers must learn more about the cellular mechanisms underlying alcohol’s toxic effects in order to develop effective approaches to reverse or prevent those effects.

## Footnotes

1 Prolactin also plays a role during fetal development and is one of the major hormones controlling the initiation and maintenance of lactation. (For more information on the functions of prolactin in lactation, see the article by Heil and Subramanian, pp. 178–184.)

2 The classification of testosterone as a “male” hormone and, similarly, of estrogen as a “female” hormone is somewhat misleading, because both hormones are present in both men and women and fulfill numerous important functions in both genders. Moreover, a portion of the testosterone in men is actually converted into estrogen. However, testosterone levels are generally higher in men than in women, and estrogen levels are generally higher in women than in men.

3 Endogenous opioids are involved in the regulation of higher brain functions, such as euphoria, pleasure, and reward. For more information on endogenous opioids and alcohol’s effects on them, see the article by Gianoulakis, pp. 202–210.

4 A standard drink is one 12-ounce can of beer or wine cooler, one 5-ounce glass of wine, or 1.5 ounces of distilled spirits.

## References

[b1-arh-22-3-195] Adams ML, Cicero TJ (1991). Effects of alcohol on β-endorphin and reproductive hormones in the male rat. Alcoholism: Clinical and Experimental Research.

[b2-arh-22-3-195] Adams ML, Forman JB, Kalicki JM, Meyer ER, Sewing B, Cicero TJ (1993). Antagonism of alcohol-induced suppression of rat testosterone secretion by an inhibition of nitric oxide synthase. Alcoholism: Clinical and Experimental Research.

[b3-arh-22-3-195] Adler RA (1992). Clinically important effects of alcohol on endocrine function. Journal of Clinical Endocrinology and Metabolism.

[b4-arh-22-3-195] Anderson RA, Willis BR, Oswald C, Zaneveld LJD (1983). Male reproductive tract sensitivity to ethanol: A critical overview. Pharmacology, Biochemistry and Behavior.

[b5-arh-22-3-195] Badr FM, Bartke A, Dalterio S, Bulger W (1977). Suppression of testosterone production by ethyl alcohol: Possible mode of action. Steroids.

[b6-arh-22-3-195] Ching M, Valenca M, Negro-Vilar A (1988). Acute ethanol treatment lowers hypophyseal portal plasma LHRH and systemic LH levels in rats. Brain Research.

[b7-arh-22-3-195] Cicero TJ, Nock B, O’Connor L, Adams ML, Sewing BN, Meyer ER (1994). Acute alcohol exposure markedly influences male fertility and fetal outcome in the male rat. Life Sciences.

[b8-arh-22-3-195] Cobb CF, Ennis MF, Van Thiel DH, Gavaler JS, Lester R (1978). Acetaldehyde and ethanol are direct testicular toxins. Surgical Forum.

[b9-arh-22-3-195] Ellingboe J, Varanelli CC (1979). Ethanol inhibits testosterone biosynthesis by direct action on Leydig cells. Research Communications in Chemical Pathology and Pharmacology.

[b10-arh-22-3-195] Emanuele MA, Hojvat S, Emanuele NV, Zelke S, Kirsteins L, Lawrence AM (1986). The effect of alcohol on quantitative and qualitative changes in luteinizing hormone (LH) in the female rat. Endocrine Research.

[b11-arh-22-3-195] Emanuele MA, Kirsteins L, Reda D, Emanuele NV, Lawrence AM (1989). In vitro effect of ethanol exposure on basal and GnRH-stimulated LH secretion from pituitary cells. Endocrine Research.

[b12-arh-22-3-195] Emanuele MA, Tentler JJ, Reda D, Kirsteins L, Emanuele NV, Lawrence AM (1990). The effect of in vitro ethanol exposure on LHRH release from perfused rat hypothalami. Endocrine Research.

[b13-arh-22-3-195] Emanuele MA, Tentler J, Emanuele NV, Kelley MR (1991). In vivo effects of acute EtOH on rat α and β luteinizing hormone gene expression. Alcohol.

[b14-arh-22-3-195] Emanuele MA, Tentler J, Halloran MM, Emanuele NV, Wallock L, Kelley MR (1992). The effect of acute in vivo ethanol exposure on follicle stimulating hormone transcription and translation. Alcoholism: Clinical and Experimental Research.

[b15-arh-22-3-195] Emanuele MA, LaPaglia N, Steiner J, Jabamoni K, Hansen M, Kirsteins L, Emanuele N (1998). Reversal of ethanol-induced testosterone suppression in peripubertal male rats by opiate blockade. Alcoholism: Clinical and Experimental Research.

[b16-arh-22-3-195] Gordon CG, Altman K, Southren AL, Rubin E, Lieber CS (1976). The effects of alcohol administration on sex hormone metabolism in normal men. New England Journal of Medicine.

[b17-arh-22-3-195] Gordon GS, Southren AL, Lieber CS (1978). The effects of alcoholic liver disease and alcohol ingestion on sex hormone levels. Alcoholism: Clinical and Experimental Research.

[b18-arh-22-3-195] Gordon GS, Southren AL, Vittek J, Lieber CS (1979). The effect of alcohol ingestion on hepatic aromatase activity and plasma steroid hormones in the rat. Metabolism.

[b19-arh-22-3-195] Gordon GS, Vittek J, Southren AL, Munnangi P, Lieber CS (1980). Effect of chronic ethanol ingestion on the biosynthesis of steroids in rat testicular homogenate in vitro. Endocrinology.

[b20-arh-22-3-195] Lloyd CW, Williams RH (1948). Endocrine changes associated with Laennec’s cirrhosis. Annals of the American Journal of Medicine.

[b21-arh-22-3-195] McCann SM, Rettori V (1996). The role of nitric oxide in reproduction. Proceedings of the Society of Experimental Biology and Medicine.

[b22-arh-22-3-195] Pajarinen J, Karhunen PJ, Savolainen V, Lalu K, Penttila A, Laippala P (1996). Moderate alcohol consumption and disorders of human spermatogenesis. Alcoholism: Clinical and Experimental Research.

[b23-arh-22-3-195] Pohl CR, Guilinger RA, Van Thiel DH (1987). Inhibitory action of ethanol on luteinizing hormone secretion by rat anterior pituitary cells in culture. Endocrinology.

[b24-arh-22-3-195] Rivier C, Vale W (1988). Interaction between ethanol and stress on ACTH and β-endorphin secretion. Alcoholism: Clinical and Experimental Research.

[b25-arh-22-3-195] Salonen I, Huhtaniemi I (1990). Effects of chronic ethanol diet on pituitary-testicular function of the rat. Biology of Reproduction.

[b26-arh-22-3-195] Shi Q, Emanuele N, Emanuele MA (1998). The effect of nitric oxide inhibitors on preventing ethanol-induced suppression of the hypothalamic-pituitary-gonadal axis in the male rat. Alcoholism: Clinical and Experimental Research.

[b27-arh-22-3-195] Southren AL, Gordon GS, Olive J, Rafii F, Rosenthal WS (1973). Androgen metabolism in cirrhosis of the liver. Metabolism.

[b28-arh-22-3-195] Steiner J, Halloran MM, Jabamoni K, Emanuele NV, Emanuele MA (1996). Sustained effects of a single injection of ethanol on the hypothalamic-pituitary-gonadal axis in the male rat. Alcoholism: Clinical and Experimental Research.

[b29-arh-22-3-195] Steiner J, Kirsteins L, LaPaglia N, Lawrence AM, Williams D, Emanuele NV, Emanuele MA (1997). The effect of acute ethanol exposure on translocation of protein kinase C in anterior pituitary. Alcohol.

[b30-arh-22-3-195] Uddin S, Wilson J, Emanuele MA, Williams D, Kelley MR, Emanuele N (1996). Ethanol-induced alterations in the posttranslational processing, but not secretion of luteinizing hormone-releasing hormone in vitro. Alcoholism: Clinical and Experimental Research.

[b31-arh-22-3-195] Van Thiel DH, Lester R, Sherins RJ (1974). Hypogonadism in alcoholic liver disease: Evidence for a double defect. Gastroenterology.

[b32-arh-22-3-195] Van Thiel DH, Lester R, Vaitukaitis J (1978). Evidence for a defect in pituitary secretion of luteinizing hormone in chronic alcoholic men. Journal of Clinical Endocrinology and Metabolism.

[b33-arh-22-3-195] Villalta J, Ballesca JL, Nicolas JM, Martinez de Osaba MJ, Antunez E, Pimentel C (1997). Testicular function in asymptomatic chronic alcoholics: Relation to ethanol intake. Alcoholism: Clinical and Experimental Research.

[b34-arh-22-3-195] Zhu Q, Van Thiel DH, Gavaler JS (1997). Effects of ethanol on rat Sertoli cell function: Studies in vitro and in vivo. Alcoholism: Clinical and Experimental Research.

